# Unveiling the Journey: A Case Report of Managing an Impacted Central Incisor

**DOI:** 10.7759/cureus.52762

**Published:** 2024-01-22

**Authors:** Anjali Kathade, Rizwan Gilani, Ranjit Kamble, Shefali Singh, Aishwarya Atey

**Affiliations:** 1 Orthodontics and Dentofacial Orthopaedics, Sharad Pawar Dental College and Hospital, Datta Meghe Institute of Higher Education and Research, Wardha, IND

**Keywords:** alignment, supernumerary tooth, surgical exposure, central incisors, impaction

## Abstract

Although impaction of the maxillary permanent central incisor is uncommon in dentistry due to its significance to facial aesthetics which are challenging to treat. To abstain from the consequences related to aesthetic and functional occlusion, early detection of an impacted central incisor is imperative. This case report describes a male patient, aged 22 years, who had an impacted central incisor tooth in the maxillary anterior region. A surgery was performed to remove the impacted supernumerary tooth that was preventing the eruption of the central incisor. Using a combination of surgical exposure and orthodontic force, the impacted right maxillary central incisor was relocated to its proper occlusion in the dental arch.

## Introduction

The appearance of the maxillary incisors when speaking and smiling greatly affects an individual's aesthetic attractiveness. Lack of an incisor can cause problems with function, especially when speaking and making sounds like "s." Therefore, both appropriate phonetics and aesthetics need the regular eruption, location, and morphology of these teeth. Between the ages of seven and nine, the mixed dentition period is when the failure of maxillary incisor eruption usually manifests itself. At an incidence ranging from 0.06% to 0.2%, this incisor is the third most often impacted tooth [1]. If the lower incisors erupt more than a year early, the contralateral incisor emerges six months earlier, or there is a deviation from the typical eruption pattern, a delayed eruption is signified.

The failure of maxillary incisor eruption can be attributed to various factors such as pathological defects, tooth deformities, an ectopic positioning of the tooth germ, tooth pulpitis or ankylosed primary teeth, disorders related to the endocrine, and bony abnormalities. The pathological obstructions can be thick tissue barriers resulting from an early extraction of the main teeth, odontomas, cysts, or additional teeth. Anterior region trauma can result in problems such as deciduous tooth loss, permanent incisor dilatation, delayed root growth, or invasive luxation. A change in the tooth's morphology or position can hinder its eruption. The extent of damage sustained by the permanent tooth is influenced by its developmental stage and the nature and direction of the trauma it experiences [2].

Early orthodontic and surgical interventions are recommended to avoid further deterioration of the tooth's alignment. Before starting orthodontic tooth correction, the impacted teeth can be exposed using a variety of surgical procedures.

## Case presentation

A male patient, aged 22 years, presented with a complaint of missing the maxillary right central incisor. The patient had no significant medical history, and his dental records revealed that a previous visit to a dental clinic had identified the impacted central incisor without receiving any treatment. Seeking more definitive care, the patient came to our department.

**Figure 1:**
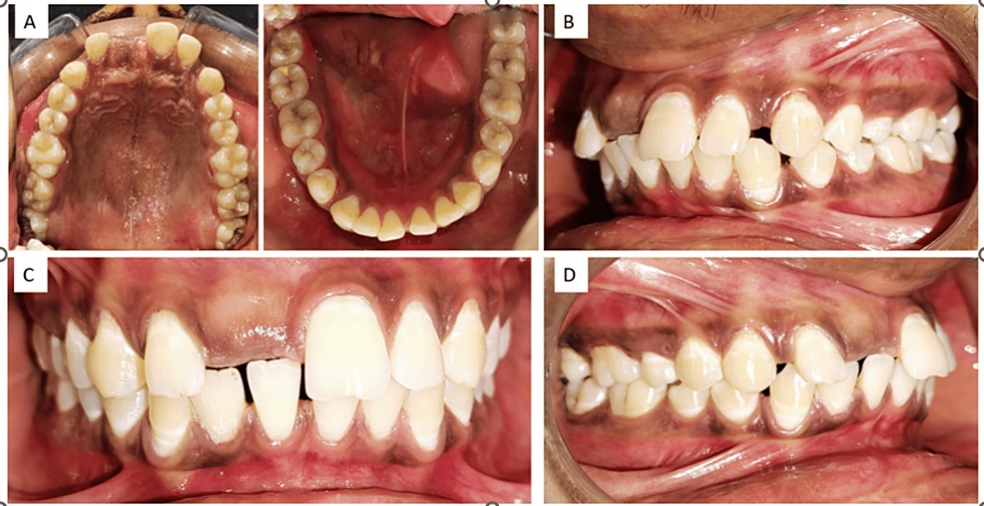
(A) Occlusal view of the maxillary arch and mandibular arch, (B) left occlusal view, (C) front occlusal view, (D) right occlusal view

**Figure 2:**
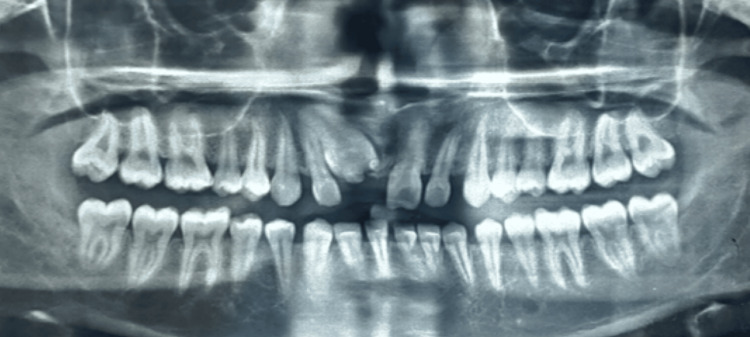
OPG Showing supernumerary teeth along with impacted central incisor in the upper right quadrant OPG: Orthopantomogram

Upon intraoral examination (Figure 1), the patient's dentition showed proclination in the anterior region with the absence of the upper central incisor. There were no signs of inflammation, infection, edema, or soreness in the affected region. To determine the location and direction of the impacted tooth, panoramic radiographs were obtained. The radiographs revealed that the impacted central incisor was positioned vertically within the maxillary bone, hindering its natural eruption (Figure 2).

**Figure 3:**
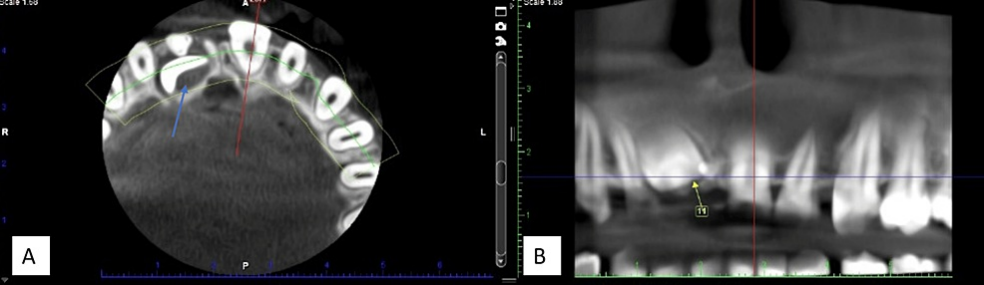
(A) CBCT Image showing supernumerary teeth with the impacted central incisor in blue arrow on axial view (B) Panoramic view showing the distance of the impacted central incisor from the alveolar crest CBCT: Cone-beam CT

The tooth could be seen protruding from the labial sulcus at the mucogingival junction, positioned in the upper part of the alveolar bone. It was surrounded by a thick layer of soft tissue and oriented vertically in the cone-beam CT (CBCT) scan (Figure 3).

**Figure 4:**
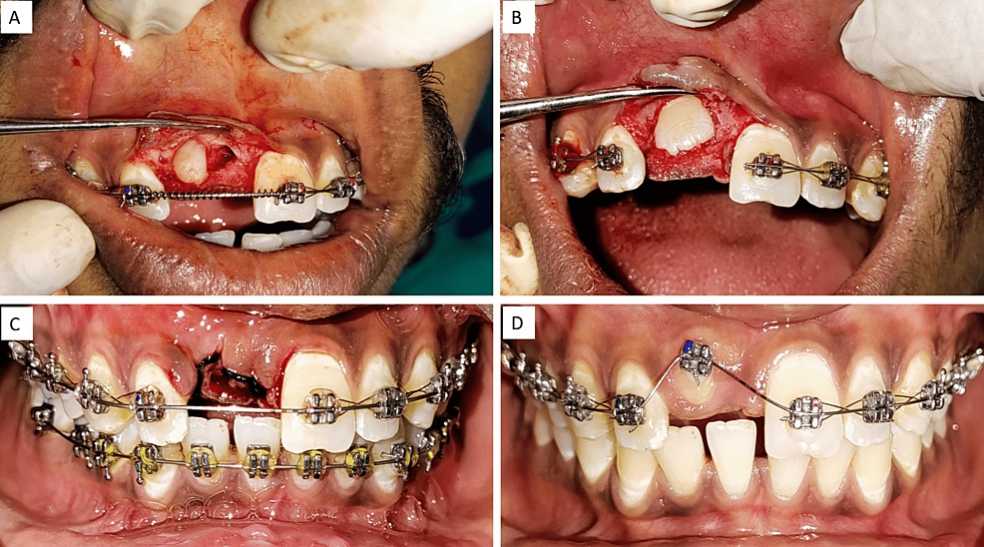
(A) Buccal flap raised to expose supernumerary teeth, (B) after removal of supernumerary tooth, a surgical exposure of the facial surface of the central incisor was done, (C) loose suture given to secure the flap, (D) image after seven days of healing

The decision was made to surgically expose the impacted tooth, and a bracket was affixed to its labial surface, guiding it into its original position. An orthodontic treatment was initiated before the surgical procedure, involving the use of 0.022*0.028 slots (McLaughlin-Bennett-Trevisi (MBT) prescription) to secure the maxillary and mandibular arches. The initial leveling and alignment were accomplished with a 0.016-inch nickel-titanium (NiTi) wire. Then a 0.0160*0.022-inch NiTi rectangular wire was placed. After achieving the initial alignment, the supernumerary tooth was extracted after surgical exposure, and the impacted right central incisor facial surface was exposed. Following this, a lingual button was initially affixed to the exposed facial surface of the central incisor, replacing the conventional bracket bonding. Traction force was applied to the impacted central incisor using a piggyback 0.016NiTi round wire, and a 0.016*0.022 stainless steel wire was the base archwire; also, teeth were consolidated with a ligature wire (Figure 4).

**Figure 5:**
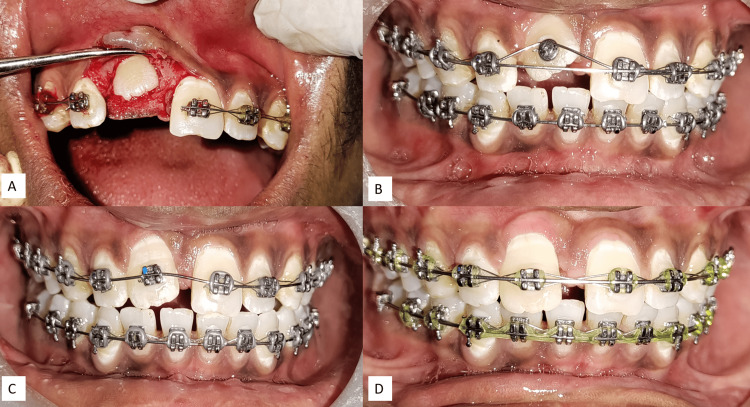
(A) After the exposure of the impacted central incisor, (B) lingual button attachment for traction, (C) complete disimpaction of the central incisor, (D) uprighting of teeth

Once the central incisor's facial position approached the occlusal plane, the central incisor bracket was then bonded to the tooth surface. After two months, a 0.017*0.025 stainless steel rectangular wire was introduced into the main slot to provide further support for the ongoing treatment. The patient had now entered the finishing and detailing stage of the orthodontic process (Figure 5). 

## Discussion

Centrally impacted teeth, sometimes referred to as impacted central incisors, pose a challenging clinical scenario in orthodontics. Impaction can result from several factors, most notably malposition, crowding, and aberrant growth patterns. Combining orthodontic and surgical treatments into one comprehensive plan is required for effective management. The main issues, difficulties, and modern methods in the orthodontic treatment of teeth with central impacted teeth are examined in this study. Developing an effective treatment plan requires a clear diagnosis. The location, angle, and proximity of the tooth to significant structures can all be determined by orthodontic and radiographic evaluations such as CBCT, cephalometric analysis, and panoramic radiography [3]. Important factors that affect how well an impacted tooth aligns include the space available for it, the degree of displacements, the orientation and location of the afflicted central incisor, and the amount of root production [4-9]. As per Lyu et al., it is imperative to examine the diverse types of impactions to determine the optimal approach for the prevention and therapy of dilacerated impacted central incisors. Depending on the crown orientation, degree of dilatation, stage of root formation, and position, teeth have different treatments and outcomes [4,5].

Early intervention in orthodontics and surgery is recommended as a preventive measure against potential future issues with tooth alignment [6]. It is feasible to detect impacted teeth through various surgical methods before initiating orthodontic tooth adjustments. The two primary surgical approaches for impacted teeth involve either the window approach, exposing the entire labial aspect and removing all keratinized tissue, or a technique that preserves 2-3 mm of keratinized tissues while exposing only the superficial 4-5 mm at the cusp tip of the labial aspect [7].

In this case, there was sufficient space for the tooth to be positioned correctly and anatomically. Studies have revealed and proved that the window approach causes a statistically significant decrease in gingival inflammation, recession, and attachment to maxillary canines after surgical exposure [8]. This method aims to create a keratinized gingiva around the tooth that is just beginning to erupt. A tooth must erupt via the gingival mucosa that is connected, not the alveolar mucosa [7-9]. If the impacted tooth is discovered to have a fully developed root system or to be in an unfavorable position, a multidisciplinary approach is required [10].

## Conclusions

In treating complicated dental conditions, this case study emphasizes the necessity of a patient-centered approach and interdisciplinary teamwork. It also emphasizes the importance of regular dental examinations and early intervention in detecting and treating dental abnormalities. The successful care of the impacted central incisor demonstrates modern dentistry's breakthroughs and the efficiency of a coordinated team effort in obtaining the best outcomes for individuals with difficult dental diseases.
